# Feed utilization efficiency and ruminal metabolites in beef cattle fed with cassava pulp fermented yeast waste replacement soybean meal

**DOI:** 10.1038/s41598-022-20471-6

**Published:** 2022-09-27

**Authors:** Gamonmas Dagaew, Sawitree Wongtangtintharn, Chanon Suntara, Rittikeard Prachumchai, Metha Wanapat, Anusorn Cherdthong

**Affiliations:** Tropical Feed Resources Research and Development Center (TROFREC), Department of Animal Science, Faculty of Agriculture, Khon Kaen University, Khon Kaen, 40002 Thailand

**Keywords:** Biochemistry, Microbiology, Zoology, Environmental sciences, Anatomy

## Abstract

The purpose of this study was to see how substituting cassava pulp fermented yeast waste (CSYW) for soybean meal (SBM) in a concentrate affected feed intake, digestibility, and rumen fermentation in Thai native beef cattle. In this study, four male Thai native beef cattle with an average age of 15.0 ± 25.0 months and body weights of 140 ± 5.0 kg were used. The experimental design was a 4 × 4 Latin squared design, with dietary treatments of CSYW replacing SBM at 0, 33, 67, and 100% in the concentrate mixture. It was discovered that the presence of CSYW had no negative impact on feed intake, nutritional intake, or apparent digestibility (p > 0.05). CSYW had no significant effects on ruminal pH or temperature (p > 0.05). When the amount of CSYW in the diet increased, the rumen ammonia–nitrogen concentration increased (p < 0.05). Blood urea nitrogen was not affected by CSYW (p > 0.05). The total bacterial population increased when the diet's CSYW amount was increased (p < 0.05). Feeding CSYW to beef cattle had no influence on total volatile fatty acid, acetic acid (C2), or butyric acid (C4) proportions (p > 0.05). The concentration of propionic acid (C3) and the C2:C3 ratio increased when the amount of CSYW in the diet was increased (p < 0.05). In conclusion, CSYW can completely replace SBM in a concentrate diet for beef cattle with no adverse effects on feed utilization or rumen fermentation while the total bacterial population and C3 concentration increase.

## Introduction

Feed resources are critical for animal productivity in general, particularly during the dry season in tropical areas. Furthermore, feed scarcity, both in terms of quality and quantity, has had a substantial impact on productivity, particularly with regard to protein sources^1^. The most popular protein source in animal diets is soybean meal (SBM), which contains about 44.0% dry matter (DM) crude protein (CP)^2^. However, the high cost of SBM in Thailand and variances in animal feed contributed to the identification of other protein sources for ruminant diets^3^. Cassava pulp is a year-round available industrial by-product of cassava starch production^4^. Cassava pulp has a CP value of 2.0–3.0% DM and a small amount of residual starch^5^, indicating that it could be utilized in ruminant animal diets. Conversely, the high fiber content of cassava pulp restricted the amount of feed consumed and the rate of digestion in cattle. Several studies have looked into how bacteria, fungus, and yeast can improve cassava pulp quality. Yeast, *Saccharomyces cerevisiae*, is the most common microbe used to improve cassava pulp quality. Sommai et al.^6^ found that using yeast-fermented cassava pulp (YFCP) instead of soybean meal was effective. YFCP’s CP content increased to 23.3% DM. Dry matter intake, rumen fermentation, nutritional digestibility, and nitrogen retention all improved significantly in Thai beef cattle fed 300 g/day of yeast-fermented cassava pulp (YFCP), while the bacterial population, protozoal population, and methane output all decreased. However, using commercial yeast products may increase feed costs. Therefore, an alternative yeast source should be considered. Yeast waste (liquid form) can now be generated from bioethanol plant factories using *Saccharomyces cerevisiae* to ferment molasses. Ethanol was the primary product, but yeast waste produced a significant amount of waste, which might pollute the environment. As a result, it seems to be possible to recycle yeast waste into animal feed. In yeast waste, 60–70% of yeast cells and 25.0–30.0% of CP are found^7,8^. As a consequence, using yeast waste to improve cassava pulp quality could be intriguing, especially in terms of lowering feed costs and reducing factory waste. Dagaew et al. ^5^ have revealed that employing yeast waste fermented cassava pulp can enhance the CP of feed products from 23.0 to 53.7% DM and can replace SBM up to 75.0% in concentrate diets without impairing gas production or in vitro digestibility. However, an in vivo experiment is required to evaluate the cassava pulp fermented yeast waste (CSYW) product. We hypothesized that CSYW is comparable to SBM in terms of feed utilization and ruminal fermentation when used as a beef cattle diet. The goal of this study was to see how feed intake, digestibility, and ruminal fermentation in Thai native beef cattle were affected by using cassava pulp fermented yeast waste instead of soybean meal in concentrate diets.

## Results

### Chemical composition of diets

Table 1 shows data on feed ingredients and chemical composition. In the current study, the CP content of the CSYW product was 53.7% DM, and the concentrations of CP in the concentrate diets were comparable among treatments, ranging from 14.2 to 14.4% DM, while the ash, NDF, and ADF were enhanced as the level of CSWY was increased.

**Table 1:** Feed ingredients and chemical composition used in the experimental ration.

Ingredients, % DM	Replacement SBM by CSYW, %				CSYW	Rice straw
	0	33	67	100		
Cassava chip	58.0	58.4	58.4	58.6		
Rice bran	12.0	12.0	12.0	12.0		
Palm kernel meal	8.0	8.0	8.0	8.0		
Soybean meal	18.0	12.1	5.9	0.0		
CSYW	0.0	5.9	12.1	18.0		
Mineral premix	0.5	0.5	0.5	0.5		
Urea	1.0	0.8	0.6	0.4		
Molasses	1.0	1.0	1.0	1.0		
Pure sulfur	1.0	1.0	1.0	1.0		
Salt	0.5	0.5	0.5	0.5		
**Chemical composition**						
Dry matter, %	90.6	90.1	90.4	91.2	35.0	92.4
**% DM**						
Organic matter	95.8	93.0	90.1	90.2	84.5	86.5
Ash	4.2	7.0	9.9	9.8	15.5	13.5
Crude protein	14.3	14.3	14.4	14.4	53.7	2.3
Neutral detergent fiber	15.0	20.7	25.8	27.2	24.3	75.5
Acid detergent fiber	9.2	12.6	17.4	18.3	11.3	55.3
Total digestible nutrient^a^	70.3	70.2	70.2	70.1	78.5	54.1

*CSYW* cassava pulp fermented yeast waste, *SBM* soybean meal.

^a^Calculated value.

### Feed utilization efficiency

Table 2 demonstrates the influences of CSYW on total intake, nutritional intake, and digestibility in Thai native beef cattle. Rice straw feed intake and total intake were unaffected by the administration of CSYW (p > 0.05). Increasing CSYW did not affect OM, CP, NDF, or ADF feed intake (p > 0.05). Furthermore, the level of substitution did not affect the apparent digestibility of DM, OM, CP, NDF, and ADF (p > 0.05).

**Table 2:** Effect of cassava pulp fermented with yeast waste (CSYW) in concentrate diets on feed intake and nutrient digestibility.

Items	Replacement SBM by CSYW, %				SEM	p-value	Contrast	
	0	33	67	100			Linear	Quadratic
**Dry meter intake**								
Concentrate								
kg/day	0.76	0.75	0.73	0.73	0.01	0.36	0.12	0.32
g/kg BW^0.75^	175.1	175.1	173.7	173.7	0.06	0.31	0.32	0.98
Roughage								
kg/day	1.8	1.8	1.9	1.8	0.05	0.85	0.11	0.97
g/kg BW^0.75^	423.4	425.0	440.7	442.6	0.87	0.51	0.23	0.54
Total								
kg/day	2.6	2.6	2.6	2.6	0.05	0.89	0.55	0.56
g/kg BW^0.75^	598.5	600.0	614.4	559.9	0.89	0.57	0.43	0.56
**Nutrients intake, kg/day**								
Organic matter	3.1	3.6	3.6	3.5	0.15	0.17	0.32	0.87
Crude protein	0.13	0.14	0.14	0.14	0.21	0.06	0.22	0.89
Neutral detergent fiber	2.0	2.0	2.0	2.0	0.01	0.39	0.88	0.93
Acid detergent fiber	1.3	1.3	1.3	1.3	0.01	0.42	0.21	0.12
**Nutrients digestibility**								
Dry matter, g/kg DM	656.1	659.3	667.8	666.7	1.71	0.72	0.23	0.98
Organic matter, g/kg DM	662.9	662.6	663.9	663.0	0.08	0.53	0.33	0.45
Crude protein, g/kg DM	609.3	609.6	653.3	642.4	2.57	0.71	0.32	0.43
Neutral detergent fiber, g/kg DM	550.7	542.2	558.1	554.0	1.90	0.23	0.11	0.89
Acid detergent fiber, g/kg DM	438.6	410.1	423.2	442.8	2.95	0.35	0.49	0.19

*CSYW* cassava pulp fermented yeast waste, *SBM* soybean meal, *SEM* standard error of the mean.

### Rumen ecology, and blood urea-nitrogen

Table 3 shows the ruminal pH, temperature, NH_3_-N concentration, and blood urea nitrogen (BUN) concentration of Thai native beef cattle given different levels of CSYW. Replacement of SBM with CSYW had no effect on ruminal pH or temperature (p > 0.05), which remained steady from 6.7 to 6.9 and 38.3 to 38.6 °C, respectively. The amount of CSYW in the concentrate diet linearly enhanced the rumen NH_3_-N concentration (p < 0.05). BUN was unaffected by the treatments (p > 0.05).

**Table 3:** Effect of cassava pulp fermented with yeast waste obtained from bioethanol processing (CSYW) in concentrate diets on ruminal pH, temperature, NH_3_-N concentration, and blood urea-nitrogen concentration of Thai native beef cattle.

Items	Replacement SBM by CSYW, %				SEM	p-value	Contrast	
	0	33	67	100			Linear	Quadratic
**Ruminal pH**								
0 h post feeding	6.9	6.8	7.0	6.9	0.09	0.57	0.11	0.22
4 h post feeding	6.8	6.6	6.7	6.7	1.09	0.41	0.21	0.43
Means	6.9	6.7	6.9	6.8	0.07	0.47	0.90	0.66
**Ruminal temperature, °C**								
0 h post feeding	38.3	38.5	38.6	38.3	0.10	0.25	0.78	0.45
4 h post feeding	38.3	37.7	38.6	38.6	0.13	0.14	0.34	0.54
Means	38.3	38.1	38.6	38.5	0.10	0.33	0.11	0.34
**NH**_**3**_**-N concentration, mg/dl**								
0 h post feeding	12.6^c^	13.0^bc^	13.3^ab^	13.6^a^	0.18	0.03	0.01	0.88
4 h post feeding	14.2^b^	14.7^b^	16.2^a^	16.5^a^	0.24	0.03	0.02	0.84
Means	13.4^b^	13.8^b^	14.7^a^	15.0^a^	0.13	< 0.01	0.05	0.12
**Blood urea-nitrogen concentration, mg/dl**								
0 h post feeding	11.3	11.4	11.6	11.8	0.14	0.19	0.11	0.98
4 h post feeding	12.3	12.2	12.2	12.2	0.31	0.99	0.34	0.08
Means	11.8	11.8	11.9	12.0	0.21	0.89	0.91	0.81

*SYW* cassava pulp fermented yeast waste, *SBM* soybean meal, *SEM* standard error of the mean.

^a–c^Value on the same row with different superscripts differ (p < 0.05).

### Ruminal microbes

The microbial population is shown in Table 4, which demonstrates that when the intake of CSYW was increased, the overall bacterial population quadratically increased in 0 h (p < 0.05). Increasing the amount of CSYW in the diet did not affect the overall protozoal population or fungus, with values ranging from 3.6 to 3.9 × 10^5^ and 2.3 to 3.0 × 10^6^, cells/ml, respectively (p > 0.05).

**Table 4:** Effect of cassava pulp fermented with yeast waste obtained from bioethanol processing (CSYW) in concentrate diets on ruminal microbes in Thai native beef cattle.

Items	Replacement SBM by CSYW, %				SEM	p-value	Contrast	
	0	33	67	100			Linear	Quadratic
**Ruminal microbes, cells/ml**								
Bacteria, × 10^12^								
0 h post feeding	3.8^b^	3.5^b^	4.8^ab^	5.6^a^	0.01	0.02	0.11	0.05
4 h post feeding	4.0	5.7	5.8	5.8	0.59	0.22	0.88	0.12
Means	3.9	4.3	5.3	5.7	0.48	0.10	0.34	0.31
Protozoa, × 10^5^								
0 h post feeding	2.5	2.5	2.6	2.5	0.10	0.82	0.54	0.49
4 h post feeding	2.5	2.6	2.7	2.6	0.04	0.44	0.89	0.80
Means	2.5	2.6	2.7	2.6	0.05	0.80	0.11	0.66
Fungi, × 10^6^								
0 h post feeding	0.2	0.2	0.3	0.2	0.62	0.55	0.12	0.32
4 h post feeding	0.2	0.2	0.2	0.3	0.38	0.52	0.91	0.78
Means	0.2	0.2	0.3	0.3	0.37	0.74	0.34	0.88

*CSYW* cassava pulp fermented yeast waste, *SBM* soybean meal, *SEM* standard error of the mean.

^a–b^Value on the same row with different superscripts differ (p < 0.05).

### Ruminal volatile fatty acid metabolites

The effect of different ratios of CSYW on the ruminal volatile fatty acid profile is shown in Table 5. SBM replacement with CSYW in Thai native beef cattle's concentrate diet had no effect on total VFA, acetic acid (C2), or butyric acid (C4) proportions (p > 0.05), which ranged from 80.4 to 81.9 mmol/l, 59.2 to 60.6, and 13.3 to 13.6 mol/100 mol, respectively. At 4 h after feeding, the concentration of propionic acid (C3) increased linearly with increasing CSYW content in the concentrate diet (p < 0.05).

**Table 5:** Effect of cassava pulp fermented with yeast waste obtained from bioethanol processing (CSYW) in concentrate diets levels on volatile fatty acid profiles in the rumen of Thai native beef cattle.

Items	Replacement SBM by CSYW, %				SEM	p-value	Contrast	
	0	33	67	100			Linear	Quadratic
**Total volatile fatty acid, mmol/l**								
0 h post feeding	73.8	74.3	76.0	75.9	1.63	0.73	0.29	0.10
4 h post feeding	86.9	87.2	87.9	88.0	0.81	0.21	0.77	0.44
Means	80.4	80.7	81.9	81.9	1.22	0.45	0.12	0.45
**Volatile fatty acid profiles, mol/100 mol**								
Acetic acid (C2)								
0 h post feeding	60.5	60.7	61.4	61.6	1.56	0.95	0.32	0.22
4 h post feeding	57.9	58.4	59.6	57.4	0.79	0.33	0.51	0.45
Means	59.2	59.6	60.6	59.4	1.05	0.79	0.89	0.71
Propionic acid (C3)								
0 h post feeding	28.1	27.4	26.4	29.9	0.80	0.12	0.66	0.47
4 h post feeding	28.6^b^	28.3^bc^	27.5^c^	29.7^a^	0.28	< 0.01	0.02	0.52
Means	28.4^ab^	27.8^b^	26.9^b^	29.8^a^	0.53	0.04	0.35	0.04
Butyric acid (C4)								
0 h post feeding	13.3	13.5	14.2	14.5	0.56	0.43	0.12	0.91
4 h post feeding	13.3	13.2	12.8	12.8	0.63	0.91	0.22	0.72
Means	13.3	13.3	13.5	13.6	0.42	0.93	0.98	0.56
C2:C3								
0 h post feeding	2.1	2.2	2.3	2.0	0.32	0.09	0.44	0.45
4 h post feeding	2.0	2.0	2.1	1.9	0.18	0.14	0.34	0.19
Means	2.0	2.1	2.2	2.0	0.24	0.25	0.80	0.42

*CSYW* cassava pulp fermented yeast waste, *SBM* soybean meal, *SEM* standard error of the mean.

^a–c^Value on the same row with different superscripts differ (p < 0.05).

## Discussions

The CSYW product contains high nutritional values, particularly nitrogen, which may be required by rumen microorganisms and animal hosts. This could be the principal consequence of the medium solution containing 50 g of urea, which resulted in a higher N content in the product. Furthermore, throughout the fermentation process, yeast containing YW may proliferate in the form of single-cell proteins, which could explain the apparent increase in CP^5^.

SBM replacement by CSYW in concentrate diets up to 100% had no adverse effects on feed utilization, such as intake and digestibility. When fermented cassava pulp with yeast waste product was added to a cattle concentrate diet, the results show that it is comparable to SBM. Although cassava pulp has a poor nutritional value, fermenting it with yeast waste might make it more palatable and digestible. Yeast waste containing molasses may promote feed intake and favor animals, increasing feed consumption^3^. The use of CSYW containing yeast cells and urea may greatly accelerate the digestion of cassava pulp. According to Suntara et al.^9^, yeast can secrete fibrolytic enzymes that can break down the fiber content in cassava pulp. Furthermore, a high urea concentration in CSYW could be another strategy to increase feed digestion. During the fermentation stage of CSYW, alkaline agents generated from urea cause the hemicellulose–lignin complex in cassava pulp to swell^10^. By chemically dissolving structural fibers' ester linkages, concentrated alkaline substances can physically inflate them. In a prior in vitro investigation^5^, discovered that when SBM was replaced with 100% CSYW, in vitro dry matter digestibility did not change. Similarly, Cherdthong and Supapong^11^ showed that feeding dairy calves a concentrate diet containing 20.0% cassava bioethanol waste fermented by *S. cerevisiae* had no negative impact on feed intake or nutrient digestion. Thus, the present study indicated that the inclusion of CSYW in the cattle diet did not lower feed utilization efficiency when compared to SBM.

The increase of CSYW up to 100% was not influenced by ruminal pH and temperature and was reported in the optimal range. This could be because the CSYW production method uses yeast waste as the primary component, resulting in a product that is high in live yeast. Yeast has been demonstrated to play an important role in the preservation of a healthy rumen environment through its association with lactate-using bacteria (LUB). Increased LUB in the rumen suppresses the activity of a lactate-producing bacterium, resulting in less lactic acid accumulation in the rumen and, as a result, the rumen pH is maintained^12^. According to Monnerat et al.^13^, supplementing yeast with a high ratio of concentrate diet to cattle maintains ruminal pH. In a similar study, Cherdthong and Supapong^11^ found that adding 20.0% YECAW to the concentrate diet did not affect ruminal pH or temperature. The pH and temperature remain constant at 38.8 and 39.0 °C, respectively. Moreover, Dagaew et al.^5^ found that CSYW replacement SBM in concentrate at 100% did not affect ruminal pH or ruminal temperature.

The concentration of NH_3_-N was increased with increasing CSYW levels. This could be because when the amount of CSYW is increased, the higher CP from CSYW is broken down by microorganisms into large quantities of NH_3_-N. However, the level of NH_3_-N remained within the normal range, indicating that it was capable of supporting beneficial microbial activity. Our results were similar to those of Amin et al.^12^ and Sommai et al.^6^, who showed that the optimum ammonia–nitrogen content in ruminal fluid for microbial development was found to be between 13.0 and 16.0 mg/dl when the dairy calf and Thai native beef cattle were fed YECAW and YFCP with rice straw as a roughage source.

A BUN concentration was also determined to investigate the association between rumen NH_3_-N and utilizing protein. According to our results, BUN did not alter between treatments. This could be due to the fact that bacteria in the rumen utilize NH_3_-N to proliferate, limiting the amount of NH_3_-N that enters the bloodstream. Normally, ruminal NH_3_-N ranges from 6.3 to 25.5 mg/dl^14^, and optimum levels (those associated with maximum performance) of plasma UN in beef cattle are reported to be between 11.0 and 15.0 m g/100 ml^15^. These results indicated that CSYW has no adverse effect on BUN concentration. Similarly, Cherdthong and Supapong^11^ found that including YECAW in the diet up to 20.0% of the time did not affect BUN concentration in dairy calves.

The concentration of C3 was increased when the level of CSYW replacement soybean meal was increased to 100%. A higher proportion of C3 in the rumen of matured cattle has been reported after yeast supplementation^12^. Yeast supplementation might affect the rumen microbial population, making the environment more favorable for many types of bacteria, resulting in an alteration in the types and amounts of individual VFA produced in the rumen. Yeast's ability to promote the growth of *M. elsdenii*, bacteria that have been shown to break down lactate into C3 and C4^16^. Sommai et al.^6^ found that the C3 was greater when supplemented with a yeast-containing product, YFCP, in concentrate diets. Supplemental YFCP in concentrate diets and supplemental YFCP at 300.0 g/day increased C3 in Thai native beef cattle to the highest level. In addition, Chen et al.^17^ found that adding yeast-fermented cassava chip protein to concentrate diets improved total VFA and C3 levels considerably while decreasing the C2:C3 ratio. Furthermore, Wanapat et al.^1^ reported that the concentrations of total VFA and C3 concentration were increased when the yeast-fermented cassava chip was supplemented.

The substitution of SBM with CSYW in a concentrated diet at 100% did not have a negative effect on feed intake, rumen fermentation, digestibility, or total bacteria populations. CSYW replacement soybean meal improved C3 concentration in Thai native beef cattle. The use of CSYW as a protein source in diets up to 100% minimizes feed costs, pollutants, and the hazards of inappropriate waste disposal.

## Materials and methods

### Animal care and experiment location

This study evaluated beef cattle management, as well as other pertinent processes, in compliance with the National Research Council of Thailand's Guidelines for the Ethics of Animal Experimentation, with authorization from the Animal Ethics Committee of Khon Kaen University, no. IACUC-KKU-108/63.

Our study confirmed that all methods were performed in accordance with the relevant guidelines and regulations. The study was carried out in compliance with the ARRIVE guidelines. During the course of the investigation, neither anesthesia nor animal sacrifice was performed. All experimental animals are animals under the supervision of the Tropical Feed Resources Research and Development Center (TROFREC), Khon Kaen University which consented to this study, and all animal samples have been collected with permission.

### Dietary preparation

The yeast waste (YW) was obtained by Khon Kaen Sugar Industry Public Co., Ltd. Cassava pulp, commercial-grade urea, and molasses were supplied by a local company. According to Dagaew et al.^5^, the following media and solutions were mixed: (1) In a flask, CSYW was mixed with 100 ml YW and 100 ml distilled water, combined, and incubated for 2 h at room temperature. (2) The medium solution was made by combining 50.0 g/kg of urea and 24.0 g/kg of molasses in 100 ml distillation water, then correcting the pH of the medium solution to 3.5–5.0 with H_2_SO_4_. (3) After mixing solutions (1) and (2) at a 1:1 ratio, they were flushed with oxygen for 18 h. (4) After 18 h, a proportion of yeast medium solution (3) was mixed with cassava pulp in a ratio of 50.0 ml to 100 g/kg; the product (5) was then fermented for 14 days before being sun-dried for 72 h. CSYW was content CP at 53.7% DM, which was thoroughly examined and incorporated into the concentrate diet in the current study. Table 1 lists the experimental diet items as well as their chemical composition.

### Animals and experimental design

The beef employed in this research originated from the Animal Farm Department of Khon Kaen University's Faculty of Agriculture. It is difficult to provide more animals for the present investigation since the Animal Farm Department has a restricted number of animals with the same condition (breed, BW, age, etc.). As a result, only a few animals were used. However, this work was conducted with caution and precision to avoid any inadvertent errors made by humans, animals, or the environment. Four male Thai native beef cattle with an average age of 15.0 ± 25.0 months and body weights of 140 ± 5.0 kg were used. The research methodology was a 4 × 4 Latin squared design, with experimental diets of CSYW substituting SBM in the concentrate combination feed at 0, 33, 67, and 100%.

### Feeding, samples collection, and laboratory analysis

Individual pens were assigned to the animals. All animals were fed 0.5% BW of concentrate and rice straw ad libitum at 7:00 a.m. and 4:00 p.m. The experiment was divided into four periods, each of which lasted 21 days. In the last 7 days of the study, animals were moved to metabolism crates to collect total feces. Feed and refusal were recorded in order to calculate daily feed intake and nutrient consumption. Nutrient digestibility was estimated using chemical composites of feces and feed samples. After drying at 60 °C, feed and feces samples were ground to pass a 1 mm sieve and measured for dry matter (DM), ash, and CP using the AOAC method^18^. In terms of organic matter, the ash percentage was subtracted from 100. The Van Soest et al.^19^ methods were utilized to test neutral detergent fiber (NDF) and acid detergent fiber (ADF) using an Ankom Fiber Analyzer (Ankom Technology). On the last day of each period, the rumen fluid and blood samples were collected. Blood (10 ml) was drawn from each cattle's jugular vein at 0 h and 4 h after feeding and stored in a test tube with EDTA. The plasma was centrifuged for 10 min at 500×*g* to extract the plasma, which was preserved at 20 °C for analysis of blood urea nitrogen (L type Wako UN, Tokyo, Japan). A sample of the rumen fluid (50 ml) was collected by the vacuum pump linked to the stomach tube at 0 h before feeding and 4 h after feeding. A pH-temperature meter was used to rapidly monitor the temperature and pH of the rumen fluid (HANNA Instruments HI 8424 microcomputer, Singapore). Two parts of rumen fluid samples were isolated. The first component, 1 ml rumen fluid, was combined with 9 ml formalin to prevent cell lysis, and then rumen microorganisms were counted using a hemocytometer under a microscope^20^. The ammonia–nitrogen (NH_3_-N) and volatile fatty acid (VFA) profiles of rumen fluid were determined in the second proportion. To prevent substance volatilization, 1 M H_2_SO_4_ was added to 45 ml rumen fluid and centrifuged at 16,000×*g* for 15 min to obtain a supernatant for NH_3_-N and VFA analysis. A spectrophotometer (UV/VIS Spectrometer, PG Instruments, London, UK) was used to evaluate the concentration of NH_3_-N present^21^. In terms of VFA concentrations, a gas chromatography system (GC2014, Shimadzu, Tokyo, Japan) with a flame ionization detector and a 25-m 0.53-mm capillary column (BPX5, SGE Analytical Science, Victoria, Australia) was used^22^.

### Statistical methods

The statistical analysis accounted for the 4 × 4 Latin square design using the GLM procedure^23^. The data were analyzed using the model below:

$${\text{Y}}_{{{\text{ijk}}}} = \, \mu \, + {\text{ M}}_{{\text{i}}} + {\text{ A}}_{{\text{j}}} + {\text{ P}}_{{\text{k}}} + \, \varepsilon_{{{\text{ijk}}}}$$

where Y is a single observation, μ is the overall mean, M is the CSYW levels (i = 1, 2, 3, 4), A is the effect of Thai native beef cattle (j = 1, 2, 3, 4), P is the period (k = 1, 2, 3, 4), and ε is the residual effect. The data are presented as mean values and standard deviations. Duncan's new multiple range tests were used to compare means, and p < 0.05 was found to be significant, suggesting a statistical difference. Orthogonal polynomials for diet responses were determined by linear and quadratic effects.

## Data Availability

The datasets generated and analyzed during the current study are available from the corresponding author on reasonable request.
